# Sustained effect of glucagon on body weight and blood glucose: Assessed by continuous glucose monitoring in diabetic rats

**DOI:** 10.1371/journal.pone.0194468

**Published:** 2018-03-20

**Authors:** Christina Pedersen, Trine Porsgaard, Maria Thomsen, Mette Marie Rosenkilde, Nikolaj Kulahin Roed

**Affiliations:** 1 Department of GLP-1 & T2D Biology, Novo Nordisk A/S, Maaloev, Denmark; 2 Faculty of Health and Medical Sciences, Department of Biomedical Sciences, University of Copenhagen, Copenhagen, Denmark; 3 Department of Insulin Pharmacology, Novo Nordisk A/S, Maaloev, Denmark; 4 Department of Modelling, Novo Nordisk A/S, Maaloev, Denmark; Max Delbruck Centrum fur Molekulare Medizin Berlin Buch, GERMANY

## Abstract

Insulin is a vital part of diabetes treatment, whereas glucagon is primarily used to treat insulin-induced hypoglycemia. However, glucagon is suggested to have a central role in the regulation of body weight, which would be beneficial for diabetic patients. Since the glucagon effect on blood glucose is known to be transient, it is relevant to investigate the pharmacodynamics of glucagon after repeated dosing. In the present study, we used telemetry to continuously measure blood glucose in streptozotocin induced diabetic Sprague-Dawley rats. This allowed for a more detailed analysis of glucose regulation compared to intermittent blood sampling. In particular, we evaluated the blood glucose-lowering effect of different insulin doses alone, and in combination with a long acting glucagon analog (LAG). We showed how the effect of the LAG accumulated and persisted over time. Furthermore, we found that addition of the LAG decreased body weight without affecting food intake.

In a subsequent study, we focused on the glucagon effect on body weight and food intake during equal glycemic control. In order to obtain comparable maximum blood glucose lowering effect to insulin alone, the insulin dose had to be increased four times in combination with 1 nmol/kg of the LAG. In this set-up the LAG prevented further increase in body weight despite the four times higher insulin-dose. However, the body composition was changed. The insulin group increased both lean and fat mass, whereas the group receiving four times insulin in combination with the LAG only significantly increased the fat mass. No differences were observed in food intake, suggesting a direct effect on energy expenditure by glucagon. Surprisingly, we observed decreased levels of FGF21 in plasma compared to insulin treatment alone. With the combination of insulin and the LAG the blood glucose-lowering effect of insulin was prolonged, which could potentially be beneficial in diabetes treatment.

## Introduction

Glucose homeostasis is tightly regulated by the two pancreatic hormones, insulin and glucagon. Insulin has been used to treat diabetes for almost 100 years, whereas the primary clinical use of glucagon is limited to the acute treatment of insulin-induced hypoglycemia. As recently reviewed [1], glucagon has a central role in the regulation of lipid metabolism, body weight, and cardiovascular health. The mechanism by which glucagon exerts these effects are still debated. Studies in rodents suggest activation of brown adipose tissue [2, 3] whereas others have observed increasing levels of fibroblast growth factor (FGF)21 [4, 5]. Regardless of mechanism, glucagon has been shown to both decrease food intake [6–8] and increase energy expenditure [9, 10]. In addition, we have recently shown that treatment with a fixed 1:23 ratio of a long-acting glucagon analog and insulin reduced the acute risk of hypoglycemia in streptozotocin (STZ) induced diabetic rats [11]. The insulin dose causing hypoglycemia was increased from 40 nmol/kg insulin alone to 160 nmol/kg insulin in the fixed ratio. Even though, the liver glycogen was found to be a limiting factor, the data indicates the possibility of achieving a protective effect of glucagon without compromising the glucose regulation provided by insulin. Thus, a pharmacological combination of insulin and glucagon could potentially result in a net positive outcome. Glucagon increases blood glucose by directly stimulating glucose output from the liver. This effect has been shown to be transient [12]. If used pharmacologically, it is relevant to investigate the pharmacodynamic profile of glucagon after repeated dosing.

The standard evaluation of glucose regulation in experimental animals is based on intermittent blood sampling. This has several limitations including restrictions to the frequency and the volume of blood sampled [13], which creates a natural limit to the number of glucose measurements from the same animals. Furthermore, it induces animal stress which influences the accuracy of the measurements since restraining of rats have been shown to affect the blood glucose level [14]. In the present study we used the HD-XG implantable device from Data Sciences International (DSI) [15] to continuously measure blood glucose directly from arterial blood for more than 20 days. Many of the currently available glucose sensors are placed subcutaneously (sc.) to measure glucose in the interstitial fluid. However, they have several limitations: 1) The glucose readings can be delayed, especially when rapid glucose changes occur [16]. 2) The glucose level can vary due to local fluctuations in blood flow [17]. 3) The sensor can cause inflammation and result in encapsulation by fibrous tissue [18]. This limits the timeframe in which the sensor can be used. To overcome these issues, the electrochemical glucose sensor used in this study was placed directly in the abdominal aorta from where it has been shown to provide continuous measurement for up to 2 months [15].

With this approach, we were able to evaluate the blood glucose lowering effect of different insulin doses with and without repeated dosing of a long-acting glucagon analog (LAG). In a separate study, we assessed the effect of the LAG on body weight and food intake after twice daily dosing for two weeks in diabetic rats.

## Materials and methods

### Compounds and formulations

The diabetic rats were treated daily with NPH insulin (Novo Nordisk A/S) until initiation of experiment. During the experiments human insulin (Novo Nordisk A/S) and a long-acting acylated glucagon analog (LAG) (Novo Nordisk A/S) were used for twice daily dosing. Both were formulated in 5 mM phosphate, 140 mM NaCl, 70 ppm polysorbate-20, pH 7.4. For binding affinity and potency on the glucagon receptor, we refer to S1 Tables.

### Experimental animals

Male Sprague-Dawley rats (325 g.) were obtained from Taconic, Lille Skensved, Denmark. The rats were acclimatized for 10 days before induction of diabetes with STZ. The rats were in a temperature- and humidity controlled room with a 12-hour dark/light cycle and had ad libitum access to food and water.

The experiment was approved by the Danish Animal Experiment Inspectorate, under Danish Ministry of Justice, and was in accordance with the Danish Animal Experimentations Act. The experiment was performed in the Laboratory Animal Facility at Novo Nordisk A/S, Maaloev, Denmark (permit number: 2017-15-0201-01175).

#### STZ-induced diabetes

On day one, pre-weighed STZ (Sigma-Aldrich) was dissolved in 0.1 M cold citric acid buffer (65 mg/ml, pH 4.5). The rats were sedated in isoflurane and diabetes was induced by injection of 65 mg/kg STZ. From day three the rats were dosed subcutaneously with NPH insulin (Novo Nordisk A/S, 600μM, 3U/rat/day). All rats became diabetic and had basal blood glucose levels above 18 mM.

#### Surgery

On day 8–10 the rats had surgery under isoflurane anesthesia. The technical details of the system and the device have been described previously [15], and a detailed surgical guideline is available from DSI [19]. Briefly, the surgical procedure started with a midline abdominal incision. The intestines were retracted to allow access to the abdominal aorta, where the sensor was placed during brief occlusion of the blood flow. The aorta was sealed by Vetbond^TM^ Tissue Adhesive (3M, St. Paul, MN) and the catheter was secured by two small fiber patch squares (DSI, St. Paul, MN). The connector part was attached to the back muscle and the reference electrode was sutured to the inside of the abdominal wall. In the end, the electronic transmitter was placed on the inside of the intraperitoneal wall by incorporating the suture rib in the closure of the abdomen. To minimize suffering, the rats received buprenorphine (Temgesic^®^, 0.05 mg/kg), caprofen (Rimadyl^®^, 5 mg/kg) and enrofloxacin (Baytril^®^, 10 mg/kg) on the day of surgery. Caprofen treatment (5 mg/kg) was continued for three days post-surgery. The rats were allowed 5–7 days of recovery before initiation of the experiment. During the experiment there were two rats per cage; a rat with an implanted sensor and a mate. All rats received the same treatment throughout the experiment.

#### Calibration of the device

The device required an initial two-point calibration against two regular blood samples with a span in blood glucose of at least 11 mM. In the diabetic rats, this was obtained by collecting blood samples before and two hours after a sc. injection of human insulin (70 nmol/kg). During the experiment single-point calibrations were performed twice weekly by collecting a basal blood sample (10 μl) from the tail vein in the morning before dosing. The blood glucose levels were evaluated using a Biosen glucose analyzer (EKF Diagnostics, Barleben, Germany).

### Study design to evaluate glucagon effect on blood glucose

A total of 10 rats had surgery. We were able to monitor blood glucose on eight rats with implanted sensors, thus two of the rats worked as back-up. All rats were dosed sc. twice daily (7 AM and 7 PM) according to Table 1. Overall the experiment consisted of two experimental periods: a “Low insulin period” and a “High insulin period”. Initially, the rats received a low dose of insulin (86 nmol/kg) for five days, followed by four days of a similar insulin dose in combination with the LAG (1 nmol/kg), before going back to the low insulin dose alone. After 2 days of insulin up-titration, the “High insulin period” was started. The only difference from the 1^st^ experimental period was a higher insulin dose (120 nmol/kg).

**Table 1:** Overview of the twice daily treatment regimen in the two experimental periods.

	Low insulin period				High insulin period		
Day	1–5	6–9	10–11	12–13	14–15	16–19	20–21
Insulin dose	86 nmol/kg	86 nmol/kg	86 nmol/kg	94–103 nmol/kg	120 nmol/kg	120 nmol/kg	120 nmol/kg
LAG dose	-	1 nmol/kg	-	-	-	1 nmol/kg	-

Shaded background highlights the periods where the LAG was added to the insulin treatment.

Body weight and food intake were measured on daily basis during both experimental periods. The food intake was evaluated as an average per cage (group size of 10 cages). The food was manually weighed and the food consumption estimated by subtracting the amount of food left from the amount of food added. When analyzing body weight we included both the rats with implanted sensors and their mates (group size of 20 rats). At the end of the experimental period the rats were anaesthetized (5% isoflurane), sensors were removed and rats were euthanized by exsanguination.

### Study design to evaluate glucagon effect on body weight and food intake

We investigated the glucagon effect on body weight and food intake further in a follow-up experiment without implanted glucose sensors. We randomly assigned 36 rats to three dosing groups with 12 rats in each group. The rats were dosed sc. twice daily for two weeks. Group 1 received insulin alone (103 nmol/kg), group 2 received a similar insulin dose (103 nmol/kg) in combination with the LAG (1 nmol/kg) and group 3 received an insulin dose (189–412 nmol/kg) to match the lowest blood glucose level in group 1. The insulin dose in group 3 was increased during the experiment according to the accumulating effect of the LAG. Body weight and food intake were measured on daily basis. To estimate body composition an echo magnetic resonance imaging (MRI) scan was performed at the beginning and the end of the experimental period. We used an EchoMRI Body Composition Analyser (EchoMRI, Houston, TX, USA) to determine lean body mass and total fat mass on all animals. The measurements were performed according to manufacturer’s instructions and as previously described [20].

On the last day of the experimental period blood glucose was measured repeatedly for 6 hours, and 300 μl blood was collected from the sublingual vein. Afterwards, the rats were anaesthetized (5% isoflurane) and liver samples were collected, frozen in liquid nitrogen, and stored at -80°C until analysis of glycogen and triglyceride content. The rats were euthanized by exsanguination during anesthesia.

#### Determination of liver glycogen content and triglycerides

Frozen liver tissue (30–40 mg) was homogenized in sodium acetate buffer (0.15 M, pH 4.9) supplemented with Triton X-100 (0.75%). The tissue homogenate was heated to 100°C for 2 min, cooled on ice and mixed thoroughly. Afterwards, the homogenate was divided in two. The part for measurement of free glucose and triglyceride concentrations was centrifuged at 9000 G for 10 min before the supernatant was collected and stored at -20°C. The other part for measurement of total glucose concentration was incubated with amyloglucosidase (Sigma-Aldrich A1602) overnight at room temperature before centrifugation and collection of the supernatant. The Cobas 6000 analyzer (Roche Diagnostics, Indianapolis, USA) was used to determine triglycerides as well as total and free glucose concentrations according to manufactures instructions. The glycogen content in the tissue samples was then calculated by subtracting the free glucose from the total glucose concentrations.

#### Determination of FGF21 and triglycerides in plasma

The blood samples, collected from the sublingual vein, were centrifuged, and plasma was stored at -20°C until analysis. Plasma triglycerides were measured on the Cobas 6000 analyzer according to manufactures instructions. Plasma FGF21 was measured with a FGF21 Mouse/Rat ELISA kit (BioVendor RD291108200R, Brno, Czech Republic) according to the provided protocol.

### Data analysis and statistics

The data collection of calibrated blood glucose values and non-calibrated activity counts was performed by the computer program, Ponemah 6.3. The raw data was exported to Microsoft Excel with 1 min logging rate, and analyzed using MatLab 2017b.

The maximum blood glucose lowering effect of the treatments was analyzed as the change from initial baseline. The initial baseline value was defined as the mean blood glucose level 45–15 minutes before dosing, and the final baseline value was defined as mean blood glucose the last 30 minutes of the dosing period (8 hours). Area over the curve (AOC) was defined as the area between the curve and the expected baseline during the dosing period, which was taken as a linear interpolation between the initial and final baseline value. Outliers were defined as events with AOC ≤ 0, as this indicates no decrease in blood glucose as expected from insulin dosing. If an animal was defined as an outlier for the majority of a treatment period it was completely excluded for the rest of the analysis. Increased variation in the response indicated loss of sensor signal and stability. The blood glucose data are shown from 0–8 hours after dosing, as no differences between the groups were observed beyond this period. Activity was analyzed as accumulated counts for the same period of time.

GraphPad Prism was used to perform two-tailed paired t-tests to assess statistical significance between two mean values. For comparison of three or more mean values, a one way analysis of variance (ANOVA) followed by Tukey’s multiple comparisons test was used. A p value < 0.05 reflects a statistically significant difference.

## Results

**Fig 1:**
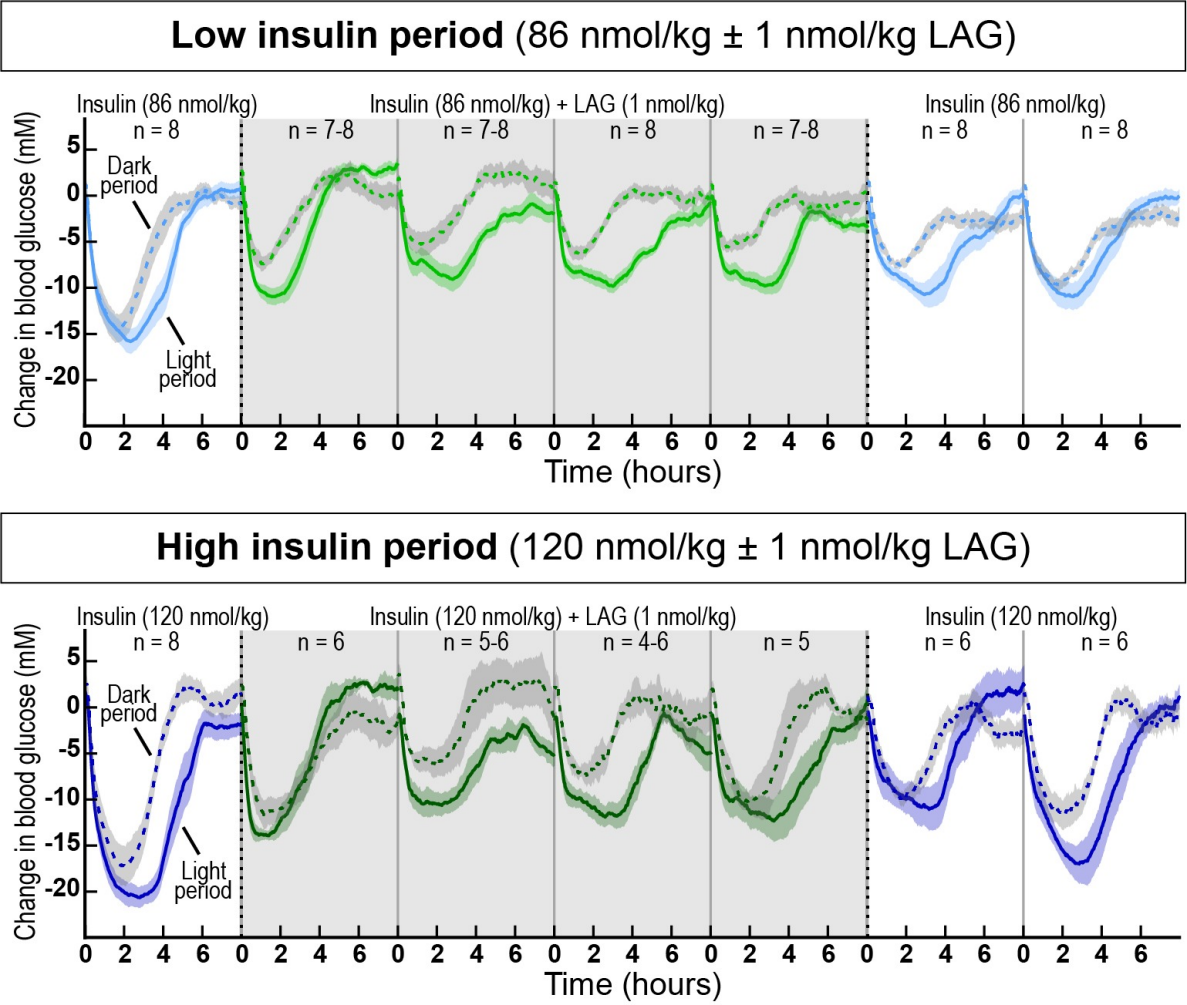
Changes in blood glucose during the two experimental periods as assessed by glucose telemetry. Solid lines represent dosing during the light periods, and dotted lines represent dosing during the dark periods. Top panel: Low insulin dose period (86 nmol/kg insulin (light blue) and 86 nmol/kg insulin + 1 nmol/kg LAG (light green)). Bottom panel: High insulin dose period (120 nmol/kg insulin (dark blue) and 120 nmol/kg insulin + 1 nmol/kg LAG (dark green)). Shaded background highlights the period where the LAG was added to the insulin treatment. Data are expressed as means ± SEM.

On day 1 of the ‘low insulin period’ and the ‘high insulin period’, the average basal blood glucose values were 25.5 ± 1.4 mM and 29.7 ± 1.3 mM, respectively. Due to this drift in basal blood glucose level during the study we used the change from baseline to compare the two dose periods. The changes in blood glucose levels during light and dark periods, measured by continuous glucose telemetry, are shown in Fig 1. Addition of 1 nmol/kg of the LAG immediately impaired the glucose lowering effect of both 86 nmol/kg (top) and 120 nmol/kg insulin (bottom). The effect was sustained after four days with twice daily dosing. When the LAG was removed from the treatment, there was still reduced effect of insulin after two days. This indicates the long-acting properties of the glucagon analog. Thus, it required several days of wash-out of the LAG to achieve the full effect of insulin.

**Fig 2:**
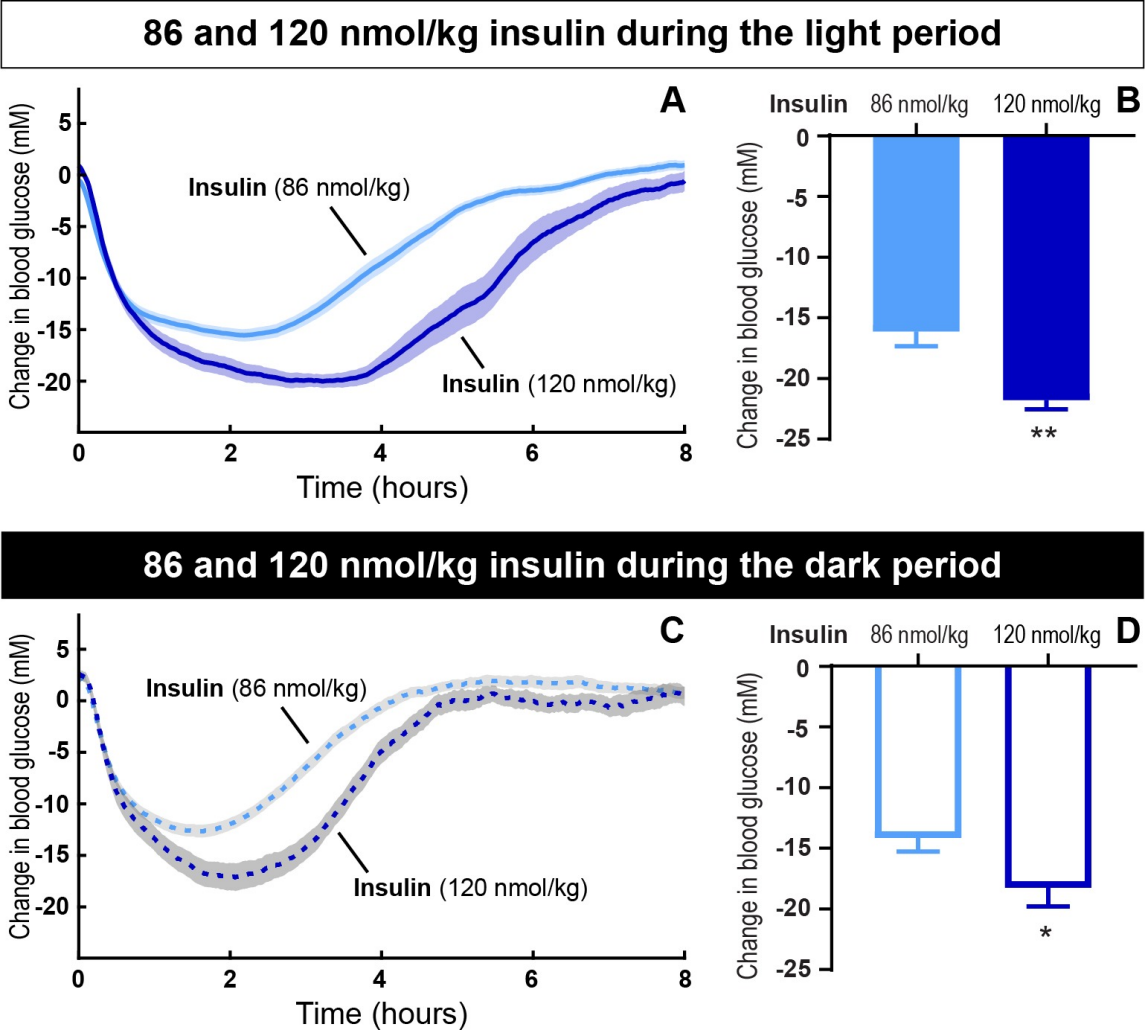
The change in blood glucose after insulin dosing during dark and light periods. The blood glucose lowering effect of 86 nmol/kg insulin compared to 120 nmol/kg insulin during light (A and B) and dark period (C and D), respectively. The change in blood glucose plotted as a function of time (A and C). The maximum change from baseline (B and D). Data are expressed as means of 5 days (86 nmol/kg) and 2 days (120 nmol/kg), respectively ± SEM; n = 7–8. A significant difference between 86 and 120 nmol/kg insulin during the light period and dark period is indicated by ** (p = 0.001) and * (p = 0.03), respectively. (analyzed using a two-tailed paired t-test).

When directly comparing the effect of the two insulin doses, 86 nmol/kg and 120 nmol/kg, there was a significant dose-dependent effect both during the light (Fig 2A and 2B, p = 0.001) and dark period (Fig 2C and 2D, p = 0.03). During the light period 86 nmol/kg and 120 nmol/kg insulin lowered blood glucose with 16.1 ± 1.2 mM and 21.8 ± 0.8 mM, respectively. During dark period 86 nmol/kg and 120 nmol/kg insulin lowered blood glucose with 14.2 ± 1.1 mM and 18.3 ± 1.5 mM, respectively. There was no significant difference between the blood glucose lowering effect of insulin during the light and the dark period for neither 86 nmol/kg nor 120 nmol/kg.

**Fig 3:**
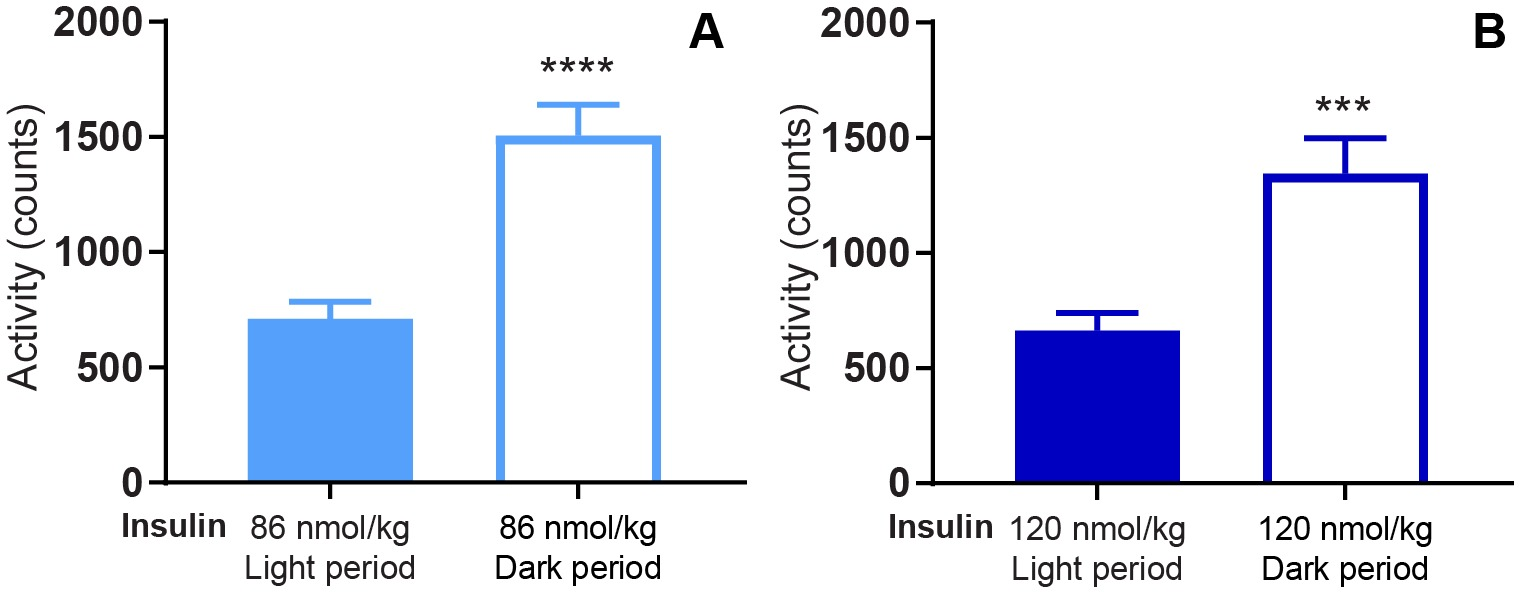
Mean activity during light and dark periods. Mean activity from 0–8 h. after dosing of 86 nmol/kg insulin (A) and 120 nmol/kg insulin (B) during both light and dark periods. Data are expressed as means of 5 days (86 nmol/kg) and 2 days (120 nmol/kg), respectively ± SEM; n = 7–8. The significant differences in activity between the light and the dark period are indicated by **** (p < 0.0001) and *** (p = 0.0003), respectively (analyzed using a two-tailed paired t-test).

Regarding activity, the rats were significantly more active during the dark period compared to the light period for both 86 nmol/kg (Fig 3A, p<0.0001) and 120 nmol/kg insulin (Fig 3B, p = 0.0003).

**Fig 4:**
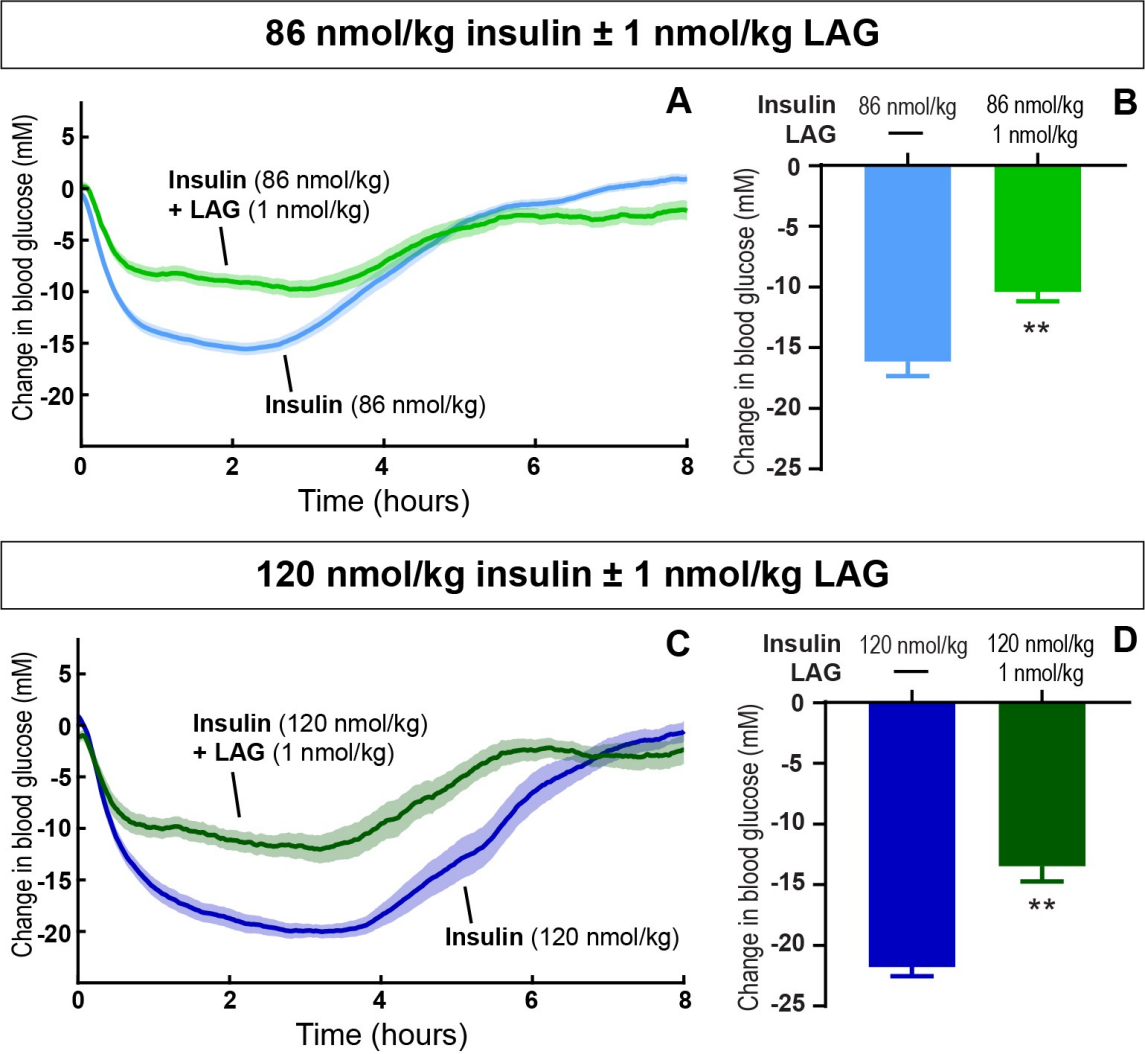
Sustained glucagon effect on blood glucose regulation. The blood glucose lowering effect of 86 nmol/kg (A and B) and 120 nmol/kg (C and D) insulin ± 1 nmol/kg LAG during the light period. The change in blood glucose plotted as a function of time (A and C). The maximum change from baseline (B and D). Data are expressed as means of 5 days (86 nmol/kg insulin) and 2 days (86 nmol/kg insulin + 1nmol/kg LAG, 120 nmol/kg ± 1nmol/kg LAG), respectively ± SEM; n = 4–8. A significant effect of adding 1 nmol/kg LAG to both 86 nmol/kg and 120 nmol/kg insulin is indicated by ** (p < 0.003) (analyzed using a two-tailed paired t-test).

In steady state (day 3 and 4 with addition of the LAG), 1 nmol/kg of the LAG significantly impaired the maximum blood glucose lowering effect of insulin during the light period. For 86 nmol/kg (Fig 4A and 4B) and 120 nmol/kg insulin (Fig 4C and 4D), the reduction was 5.7 mM (from 16.1 ± 1.2 to 10.4 ± 0.8 mM, p = 0.003) and 8.3 mM (from 21.8 ± 0.8 mM to 13.5 ± 1.3 mM, p = 0.003), respectively.

**Fig 5:**
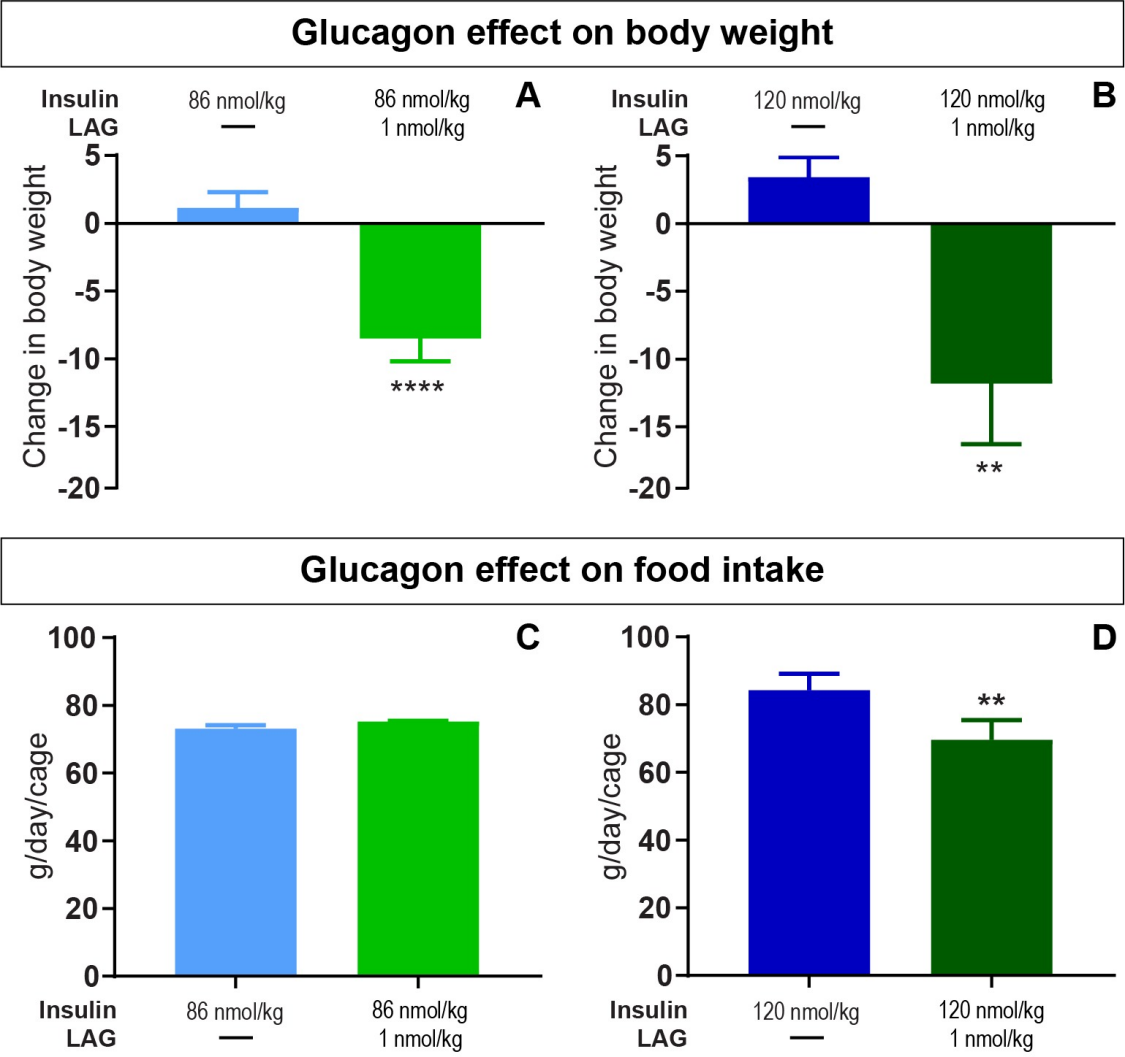
Glucagon effect on body weight and food intake. Top panel: the change in body weight after twice daily dosing for two days with insulin alone or four days in combination with the LAG. A) 86 nmol/kg insulin ± 1nmol/kg LAG. B) 120 nmol/ kg insulin ± 1nmol/kg LAG. Data are expressed as means ± SEM; n = 20. A significant change in body weight compared to insulin alone is indicated by ** (p = 0.002) and **** (p < 0.0001) asterisks (analyzed using two-tailed paired t-test). Bottom panel: The average food intake per day per animal during insulin treatment alone or in combination with the LAG. C) 86 nmol/kg insulin ± 1nmol/kg LAG. D) 120 nmol/ kg insulin ± 1nmol/kg LAG. Data are expressed as means of 2 days ± SEM; n = 10. A significant effect of adding 1 nmol/kg LAG to 120 nmol/kg insulin is indicated by ** (p = 0.001) (analyzed using a two-tailed paired t-test).

Since glucagon is known to have a potential weight-lowering effect, we monitored body weight and food intake on daily basis. As indicated on Fig 5, four days of twice daily treatment with the LAG in combination with both 86 nmol/kg insulin (Fig 5A) and 120 nmol/kg insulin (Fig 5B) significantly reduced the body weight by 8.5 ± 1.7 g. (p < 0.0001) and 11.8 ± 3.4 g. (p = 0.002), respectively. The reduction in bodyweight was not significantly different between the two insulin doses in combination with the LAG. For 86 nmol/kg insulin in combination with the LAG, the reduction in body weight was obtained without any difference in food intake (Fig 5C), whereas the food intake was significantly reduced for 120 nmol/kg insulin in combination with the LAG (Fig 5D, p = 0.001).

**Fig 6:**
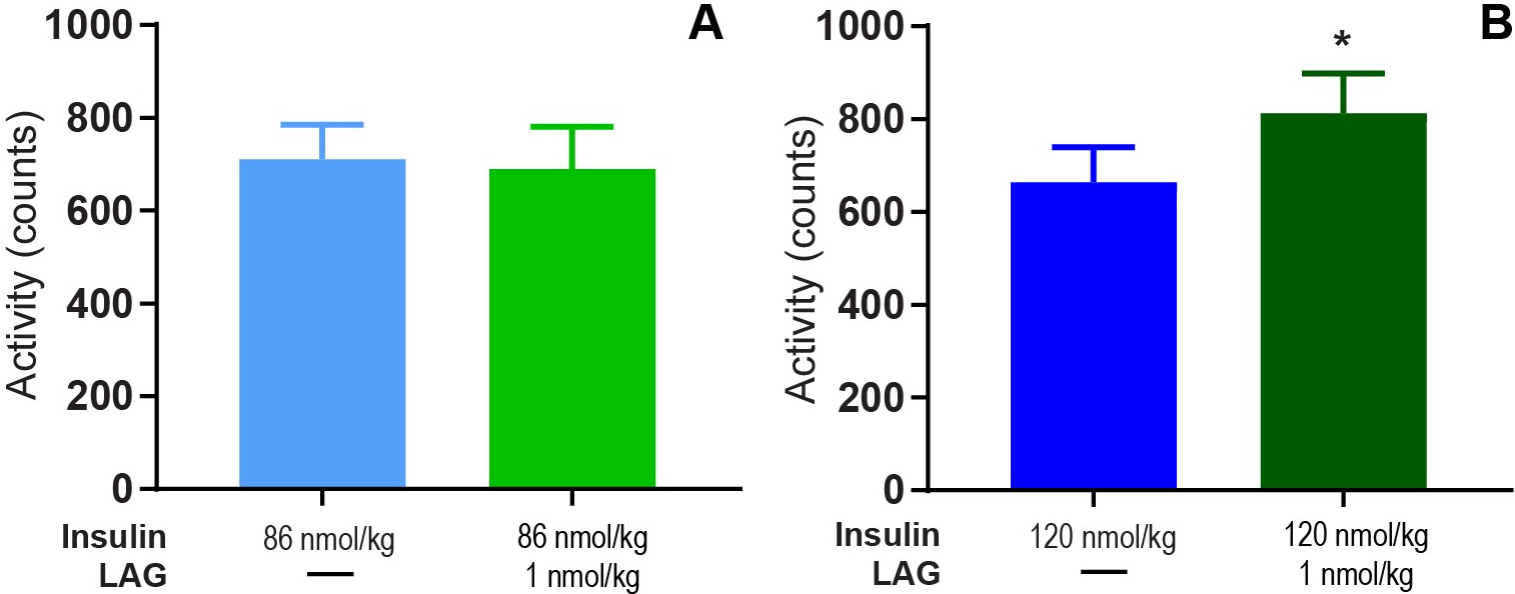
Glucagon effect on activity during light period. Mean activity from 0–8 h. after dosing of 86 nmol/kg insulin ± 1 nmol/kg LAG (A) and 120 nmol/kg insulin ± 1nmol/kg LAG (B) during the light period. Data are expressed as means of 5 days (86 nmol/kg insulin) and 2 days (86 nmol/kg insulin + 1nmol/kg LAG, 120 nmol/kg ± 1nmol/kg LAG), respectively ± SEM; n = 4–8. A significant difference in activity between 120 nmol/kg insulin and 120 nmol/kg insulin + 1 nmol/kg LAG is indicated by * (p = 0.026) (analyzed using a two-tailed paired t-test).

During the light period, we observed significantly increased activity after adding 1 nmol/kg LAG to 120 nmol/kg insulin (Fig 6B, p = 0.026). No difference was observed neither between 86 nmol/kg insulin with and without 1 nmol/kg LAG (Fig 6A), nor during the dark period for any of the doses (S1 Fig).

**Fig 7:**
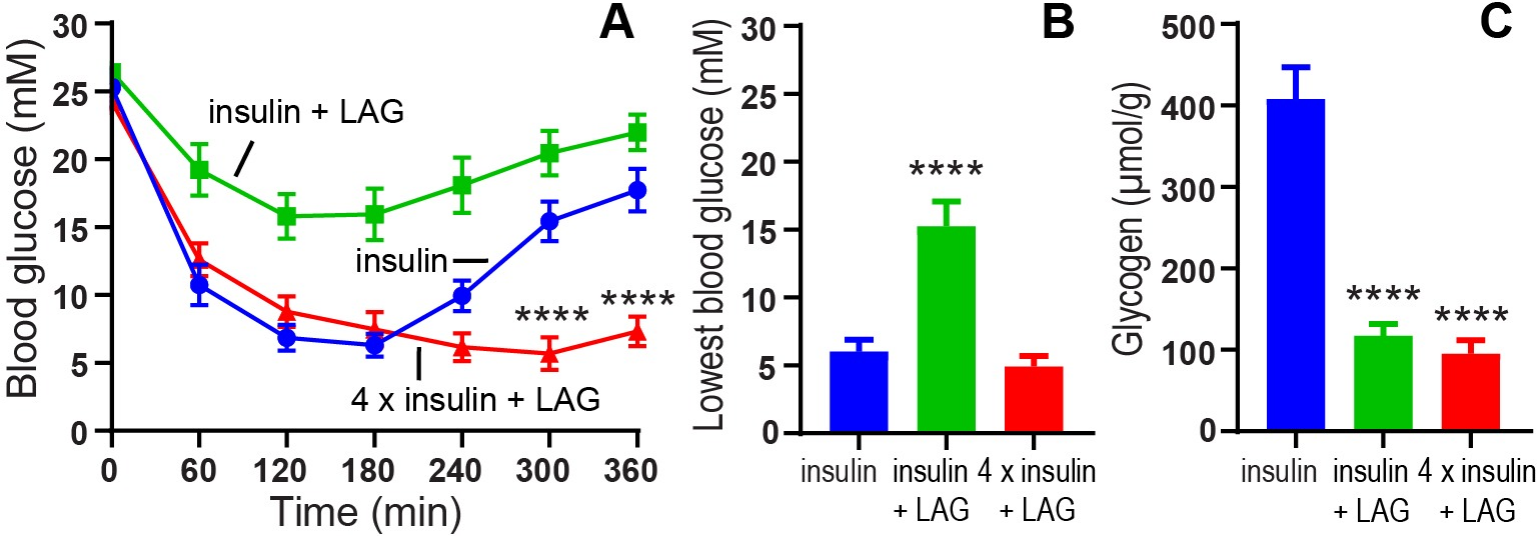
Glucagon effect on blood glucose and glycogen content in the liver. A) Blood glucose (mM) plotted as function of time. At t = 0 min. the animals were dosed sc. with insulin (103 nmol/kg), insulin (103 nmol/kg) + LAG (1 nmol/kg) or 4 x insulin (412 nmol/kg) + LAG (1 nmol/kg). Blood glucose was measured in tail tip blood for 6 hours. Significant lower blood glucose levels with 4 x insulin + LAG after 300 and 360 min are indicated by **** (p < 0.0001), (analyzed using two-way ANOVA followed by Tukey’s multiple comparisons test). B) The lowest blood glucose levels (mM) obtained during the 6-hour experiment. C) Liver glycogen content (μmol/g tissue) 6 hours after sc. dosing. Data are expressed as means ± SEM; n = 12. A significant impaired blood glucose lowering effect of 103 nmol/kg insulin + 1 nmol/kg LAG (B), and a significant increased glycogen content in the liver (C) are indicated by **** (p < 0.0001), (analyzed using one-way ANOVA followed by Tukey’s multiple comparisons test).

To further investigate the long term glucagon effect on body weight, we designed a follow-up experiment where the rats were dosed sc. twice daily for two weeks. These rats did not have implanted glucose sensors, but we measured the blood glucose manually to confirm the sustained effect of the LAG after two weeks (Fig 7A). Insulin alone (103 nmol/kg) lowered blood glucose to a minimum of 6.0 ± 0.9 mM glucose during the first 6 h. after dosing. Addition of 1 nmol/kg of the LAG significantly (p < 0.0001) impaired the blood glucose lowering effect of 103 nmol/kg insulin to 15.3 ± 1.8 mM glucose (Fig 7B). In order to normalize maximum blood glucose lowering effect in addition of the LAG, we included a group that received four times the insulin dose (412 nmol/kg) in combination with 1 nmol/kg of the LAG. In this group, the blood glucose was lowered to 4.9 ± 0.8 mM. There was no difference in the lowest blood glucose values between 103 nmol/kg insulin (blue curve) and 412 nmol/kg insulin in combination with the LAG (red curve) (Fig 7B). When comparing the shape of the curves for insulin alone (blue curve), and four times insulin in the presence of 1 nmol/kg LAG (red curve), the latter one is more flat indicated by significant lower blood glucose levels after 300 and 360 min (Fig 7A). This indicated a prolonged effect of insulin. In order to determine the glucagon stimulated glycogenolysis, liver samples were collected at the end of the experiment (6 hours after last dosing) and the amount of glycogen was determined. The amount of glycogen in the liver was significantly (p <0.0001) reduced in the two groups receiving 1 nmol/kg LAG in combination with insulin compared to insulin alone (Fig 7C). The amount of glycogen was 408 ± 39 μmol/g in the rats treated with insulin alone, compared to 117 ± 14 μmol/g in the group treated with insulin in combination with 1 nmol/LAG, and 95 ± 16 μmol/g in the group treated with four times insulin in combination with 1 nmol/kg LAG.

**Fig 8:**
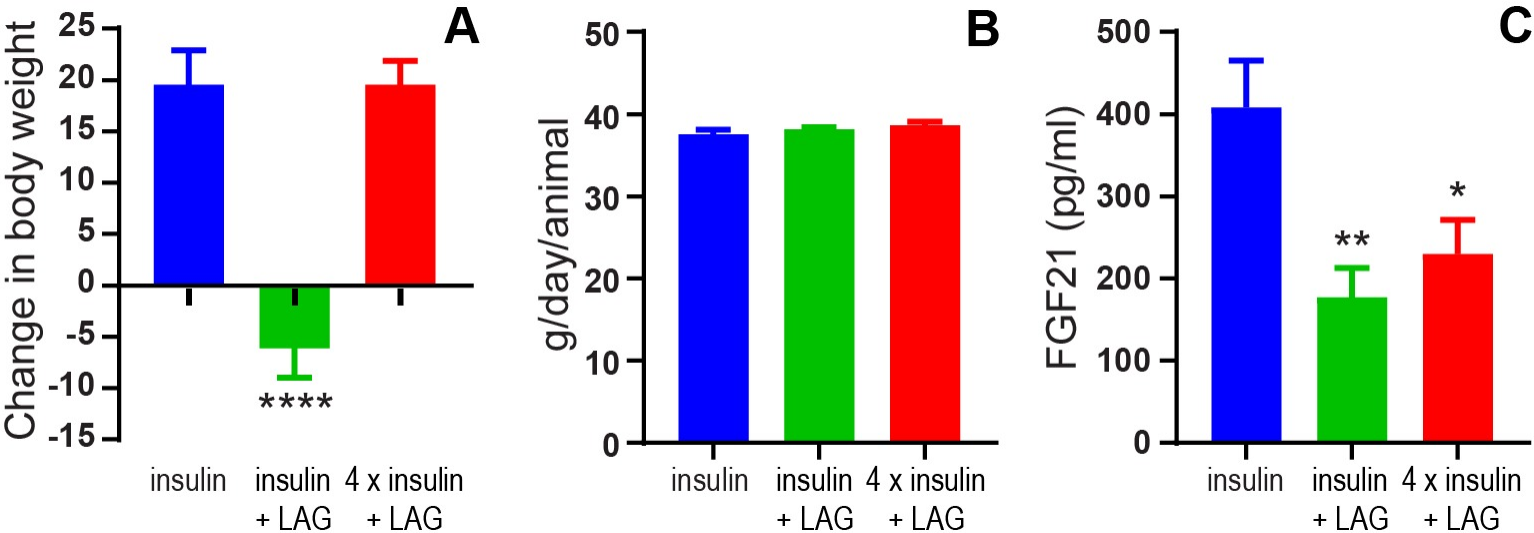
Glucagon effect on body weight, food intake and FGF21. A) The change in body weight after twice daily dosing for two weeks with insulin (103 nmol/kg), insulin (103 nmol/kg) + LAG (1 nmol/kg) or 4 x insulin (412 nmol/kg) + LAG (1 nmol/kg). B) Average food intake per animal. C) Plasma FGF21(pg/ml). Data are expressed as means ± SEM; n = 12. The significant effects of the LAG in combination with insulin or 4 x insulin, are indicated by **** (p < 0.0001), ** (p = 0.009), * (p = 0.03), respectively (analyzed using one-way ANOVA followed by Tukey’s multiple comparisons test).

The ability of the LAG to reduce body weight in combination with insulin (103 nmol/kg) was confirmed (Fig 8A). However, when the LAG was combined with a four times increased insulin dose (412 nmol/kg) the body weight increased to the same extent as with 103 nmol/kg insulin alone. These differences in body weight were not due to differences in food intake (Fig 8B). It has been suggested that glucagon increases FGF21 and thereby increases energy expenditure (4). However, compared to insulin treatment alone (103 nmol/kg), we observed significantly decreased plasma FGF21 in the groups treated with 1 nmol/kg of the LAG in combination with insulin (103 nmol/kg, p = 0.009) and four times insulin (412 nmol/kg, p = 0.03), respectively (Fig 8C).

**Fig 9:**
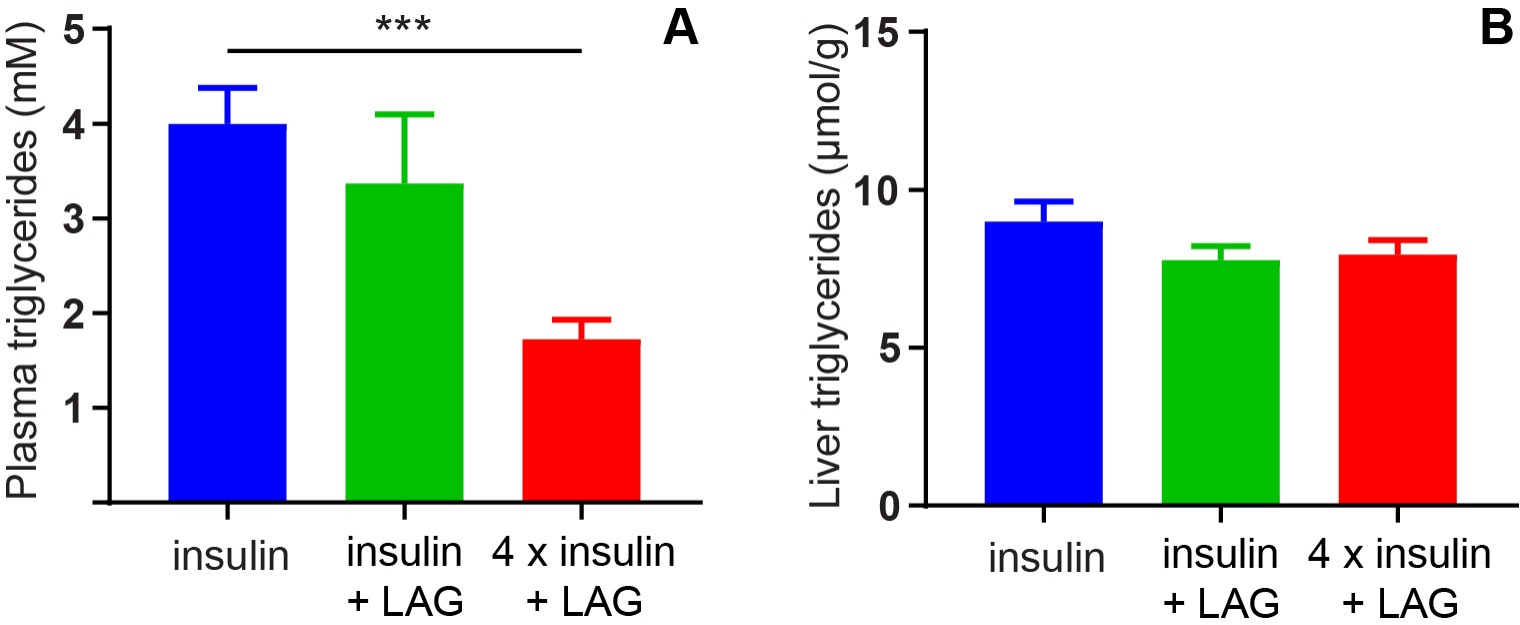
Triglycerides in plasma and liver tissue. Triglycerides in plasma (A) and liver tissue (B) after twice daily dosing for two weeks with insulin (103 nmol/kg), insulin (103 nmol/kg) + LAG (1 nmol/kg) or 4 x insulin (412 nmol/kg) + LAG (1 nmol/kg). Data are expressed as means ± SEM; n = 12. *** (p = 0.0002) indicate significant effect of the 4 x insulin + LAG compared to insulin alone (analyzed using one-way ANOVA followed by Tukey’s multiple comparisons test).

Insulin is known to stimulate lipogenesis [21]. This was indicated by significantly reduced plasma levels of triglycerides after twice daily treatment for two weeks with four times insulin (412 nmol/kg) in combination with the LAG (1 nmol/kg) compared to insulin treatment (103 nmol/kg) alone (Fig 9A). Triglyceride levels in plasma were 4.0 ± 0.4 mM in the insulin group compared to 1.7 ± 0.2 mM in the group receiving four times insulin in combination with the LAG. No significant differences were observed in liver triglycerides (Fig 9B).

Analysis of the body composition (Table 2) revealed that the insulin group (103 nmol/kg) increased body weight by significantly increasing both lean mass (p = 0.001) and fat mass (p = 0.03), whereas the group receiving four times insulin in combination with the LAG only significantly increased the fat mass (p < 0.0001). When looking at the changes in fat and lean mass normalized to percentage of body weight it also appeared that the weight gain in the 4 x insulin group was associated with more fat mass gain and less lean mass gain compared to the insulin alone group.

**Table 2:** Fat mass and lean mass at the beginning and the end of the study.

		Insulin 103 nmol/kg	insulin + LAG 103 nmol/kg + 1 nmol/kg	4 x insulin + LAG 412 nmol/kg + 1 nmol/kg
**Fat mass (g)** Average ± SEM	Start	23.2 ± 1.2 (7%)	24.0 ± 1.5 (7%)	25.2 ± 1.0 (7%)
	End	27.1 ± 1.9* (7%)	25.1 ± 1.5 (7%)	33.6 ± 1.4**** (9%)
**Lean mass (g)** Average ± SEM	Start	281.4 ± 6.3 (80%)	280.2 ± 5.0 (81%)	283.3 ± 5.8 (81%)
	End	302.8 ± 9.2*** (82%)	273.1 ± 4.9 (81%)	288.9 ± 6.1 (79%)

* indicate p < 0.05

*** indicate p < 0.001

**** indicate p < 0.0001, analyzed using paired t-test for each group

## Discussion

### The glucose telemetry technique

In the present study, we used telemetry to continuously measure blood glucose and activity for more than 20 days. The advantages of this technique include long-term and consecutive data collection to easily evaluate dose-response relationships. In the current setup, we evaluated the effect of different insulin doses with and without repeated dosing of the LAG. In particular, we showed how the effect of the LAG persisted over time (Fig 1). This had not been possible with intermittent blood sampling. Furthermore, intermittent blood sampling is more laborious and associated with animal stress. Implantable glucose telemetry allows blood glucose measurements in fully conscious and free-moving animals. However, there are also some drawbacks of the method. First of all, the required surgery and the cost of the devices make it less appropriate for high-throughput screening of compounds. Second, the need for twice weekly calibration means that it does not completely remove the need for manual blood sampling. Third, the data handling is massive. Both the data-reduction in Ponemah, and the following analysis in Excel and/or MatLab. Nevertheless, this method allowed for a more detailed analysis of blood glucose regulation compared to intermittent blood sampling. Thus, we were able to measure a dose-dependent effect of insulin during both light and dark periods (Fig 2).The measurement of activity confirmed that rats were more active during the dark period (Fig 3). Furthermore, the measure of activity added a novel aspect to the evaluation of the body weight lowering effect of glucagon (Fig 6). Even though the rats were more active during the dark period, and are expected to increase feeding in this period, we found no significant difference between the blood glucose lowering effect of insulin during the light and the dark period for any of the insulin doses. When adding 1 nmol/kg of the LAG, the glucose-lowering effect of insulin was impaired (Fig 1). Based on insulin exposure data (S2 Fig), we can exclude that the presence of the LAG affect the insulin levels in plasma. The effect is more likely due to an increased glucagon-stimulated hepatic glucose production [22, 23]. In addition, acute administration of glucagon has been shown to induce insulin resistance [24], which would also result in an impaired glucose-lowering effect of insulin. A similar effect is seen in individuals with T2D where elevated plasma glucagon levels contribute to hyperglycemia [25, 26].

A general consideration, when performing long-term studies in diabetic animals, is disease progression. In this experiment, the rats became more diabetic over time, which made it challenging to compare the glucose measurements between the two experimental periods in absolute values. To overcome this issue, we used the change in blood glucose from baseline.

### Decreased body weight without changes in food intake

We have shown that 1 nmol/kg of the LAG reduced body weight in combination with both 86 and 120 nmol/kg insulin (Fig 5A and 5B). In combination with 86 nmol/kg insulin this was obtained without any differences in food intake (Fig 5C) or activity (Fig 6A), suggesting a possible effect on enhanced energy expenditure by glucagon. However, in combination with 120 nmol/kg insulin we observed decreased food intake (Fig 5D) together with increased activity (Fig 6B) compared to insulin alone. This could potentially explain the decreased body weight. One explanation for the observed differences between the two experimental periods could be the fact of external influences. Especially activity is affected by sounds or other disturbances in the room. To account for this, a cross-over design between the two experimental periods would have been more appropriate. In the follow-up experiment, with twice daily dosing for two weeks, all treatments were given to the animals within the same period. Thereby, we avoided uncertainty whether external differences influenced the results. The reduction in body weight (Fig 8A) was obtained without changes in food intake (Fig 8B), and thus supports the tendency observed in the first experiment. Comparable results on body weight and food intake have been seen in a study with chronic glucagon-treatment of obese zucker rats for 14 months [27]. Even though, we did not measure metabolic rate it is very likely that the glucagon effect on body weight is due to increased energy expenditure. In rats, it has previously been shown that glucagon enhances metabolic rate through activation of brown adipose tissue [2, 3]. In humans, several studies support that glucagon increases energy expenditure [9, 28, 29], but this seems to be independent of activation of brown adipose tissue [10]. It has also been suggested that glucagon controls energy and lipid metabolism via FGF21-dependent pathways [4, 5]. However, after twice daily dosing for two weeks with insulin in combination with the LAG we observed decreased plasma FGF21 levels compared to insulin treatment alone (Fig 8C). This indicates that circulating FGF21 was not involved in the observed body weight lowering effect of glucagon. A similar conclusion was made after a study with 13 hours of hyperglucagonemia where they observed increased energy expenditure without increased FGF21 [9]. Whether brown adipose tissue or FGF21 is involved or not, there is a general agreement that glucagon has a positive effect on body weight reduction in both rodents and humans.

### Changed body composition and lipid profile

In order to obtain a comparable blood glucose lowering effect of insulin alone and in combination with the LAG, the insulin dose had to be increased four times in combination with 1 nmol/kg LAG (Fig 7B). When the LAG was combined with this increased insulin dose (412 nmol/kg) we observed no difference in body weight compared to the effect of the lower insulin-dose (103 nmol/kg) alone (Fig 8A). In general insulin is an anabolic hormone [30] and is well known to cause weight gain [31]. Assuming, the weight gain is proportional to the insulin dose then addition of the LAG prevents further increase in body weight with the four times higher insulin-dose. However, it is important to note the difference in body composition (Table 2). The insulin group (103 nmol/kg) increased both lean and fat mass, whereas the group receiving four times insulin in combination with the LAG only significantly increased the fat mass. This is in line with a clinical study in type 2 diabetic patients where the increase in body weight, after 6 months of insulin therapy, was exclusively due to fat mass with no improvement in muscle strength [32]. When comparing glycogen content in the liver 6 hours after last dosing, we observed a significant reduction in the group receiving 412 nmol/kg insulin in combination with the LAG compared to 103 nmol/kg insulin alone (Fig 7C). This indicates glucagon-stimulated glycogenolysis and thereby increased glucose output from the liver. Since the blood glucose was lowered to the same degree in the two groups (Fig 7B), it indicates a redistribution of the glucose from the liver to the muscle and adipose tissue. This could also explain the increased fat mass observed in the group receiving four times insulin in combination with the LAG. Since weight gain in diabetes has been shown to adversely affect cardiovascular risk [33], this has to be considered as a drawback of combining insulin and glucagon for the treatment of diabetes. On the other hand, we observed a more flat profile of the combination of insulin (412 nmol/kg) and the LAG (1 nmol/kg) compared to insulin alone (Fig 7A). This would be very beneficial in diabetes treatment as it would result in a prolonged effect of insulin, and thereby improved glycemic control. In this study, the prolonged effect of insulin led to reduced plasma triglycerides (Fig 9A), which could be positive regarding cardiovascular risk [34]. However, there are several limitations of the study to fully clarify the clinical benefits. For instance, these studies were performed on STZ-induced diabetic rats, which is considered a type I diabetic animal model. It will be relevant to address the potential anti-obesity effect of the LAG in a type II diabetic animal model. Furthermore, several lines of evidence indicate that excess glucagon plays a pathophysiological role in the development of diabetes [35]. This is further supported by studies showing beneficial effect of inhibiting glucagon secretion [36, 37] or action [38, 39]. Thus, additional studies are needed to fully assess whether a pharmacological combination of insulin and glucagon could be beneficial in diabetes treatment.

## Conclusion

By the use of continuous glucose monitoring we were able to show that the LAG had a sustained effect on blood glucose regulation after repeated dosing. Furthermore, the LAG contributed to body weight reduction without affecting food intake, which suggests a direct effect of the LAG on energy expenditure. However, this requires greater exploration. When the LAG was combined with a four times increased insulin dose, the glucose lowering effect of insulin was prolonged, which could potentially be beneficial in diabetes treatment. Furthermore, the prolonged effect of insulin led to reduced plasma triglycerides, which is positive regarding cardiovascular risk.

## Supporting information

S1 Tables Binding affinity and potency of the LAG compared to native glucagon. The binding affinity of native glucagon and the long-acting glucagon analog (LAG) to the rat glucagon receptor (rGCGR) and human GLP-1 receptor (hGLP-1R) was determined on membranes from BHK cells overexpressing the rGCGR and hGLP-1R, respectively. There was no significant difference between native glucagon and the LAG on the rGCGR (p = 0.7, analyzed using a two-tailed unpaired t-test). Data are expressed as means ± SEM.
The potency of the LAG and native glucagon was determined in primary rat hepatocytes, using the Adenylyl Cyclase Activation Flashplate® Assay. There was no significant difference between native glucagon and the LAG (p = 0.8, analyzed using a two-tailed unpaired t-test). Data are expressed as means ± SEM.
(PDF)

S1 Fig Glucagon effect on activity during dark period. Mean activity from 0–8 h. after dosing of 86 nmol/kg insulin ± 1 nmol/kg LAG (A) and 120 nmol/kg insulin ± 1 nmol/kg LAG (B) during the dark period. Data are expressed as means of 5 days (86 nmol/kg insulin) and 2 days (120 nmol/kg insulin), respectively ± SEM; n = 4–8.
(PDF)

S2 Fig Insulin in plasma with and without 1 nmol/kg LAG. Insulin in plasma (nM) 60 min after sc. dosing of 103 nmol/kg insulin ± 1 nmol/kg LAG. Insulin in plasma was measured by an internal developed LOCI assay (see details below). There was no significant difference between the plasma levels of insulin with or without the presence of 1 nmol/kg LAG (p = 0.6, analyzed using a two-tailed unpaired t-test). Data are expressed as means ± SEM; n = 12.
(PDF)

S1 File Data set 1. Experiment (continuous blood glucose measurements).
(XLS)

S2 File Data set 1. Experiment (body weight and food intake).
(XLSX)

S3 File Data set 2. Experiment.
(XLSX)
