# Supplementary material for: Sustained effect of glucagon on body weight and blood glucose: Assessed by continuous glucose monitoring in diabetic rats
Source: PLoS One. 2018 Mar 20;13(3):e0194468. doi: 10.1371/journal.pone.0194468 (PMC5860770; doi:10.1371/journal.pone.0194468)
Supplement: S1 Tables — The binding affinity of native glucagon and the long-acting glucagon analog (LAG) to the rat glucagon receptor (rGCGR) and human GLP-1 receptor (hGLP-1R) was determined on membranes from BHK cells overexpressing the rGCGR and hGLP-1R, respectively. There was no significant difference between native glucagon and the LAG on the rGCGR (p = 0.7, analyzed using a two-tailed unpaired t-test). Data are expressed as means ± SEM. The potency of the LAG and native glucagon was determined in primary rat hepatocytes, using the Adenylyl Cyclase Activation Flashplate® Assay. There was no significant difference between native glucagon and the LAG (p = 0.8, analyzed using a two-tailed unpaired t-test). Data are expressed as means ± SEM. (PDF) [file pone.0194468.s001.pdf]

**Binding affinity of native glucagon and the LAG on the rGCGR and hGLP-1R**		
	rGCGR<br>pLogIC$_{50}$ ± SEM	hGLP-1R<br>pLogIC$_{50}$ ± SEM
Native glucagon	9.4 ± 0.3 (n = 13)	< 6.0
LAG	9.6 ± 0.4 (n = 5)	Not tested

**Potency of native glucagon and the LAG in rat hepatocytes**	
	rGCGR<br>plogEC$_{50}$ ± SEM
Native glucagon	8.7 ± 0.6 (n = 5)
LAG	8.9 ± 0.2 (n = 5)

S1 Tables: Binding affinity and potency of the LAG compared to native glucagon
The binding affinity of native glucagon and the long-acting glucagon analog (LAG) to the rat glucagon receptor (rGCGR) and human GLP-1 receptor (hGLP-1R) was determined on membranes from BHK cells overexpressing the rGCGR and hGLP-1R, respectively (see details below). There was no significant difference between native glucagon and the LAG on the rGCGR ($p = 0.7$, analyzed using a two-tailed unpaired t-test). Data are expressed as means ± SEM.
The potency of the LAG and native glucagon was determined in primary rat hepatocytes, using the Adenylyl Cyclase Activation Flashplate® Assay (see details below). There was no significant difference between native glucagon and the LAG ($p = 0.8$, analyzed using a two-tailed unpaired t-test). Data are expressed as means ± SEM.

Methods

Binding affinity was determined by the scintillation proximity assay (SPA). Solubilized membranes were prepared from BHK-cells overexpressing the rGCGR and hGLP-1R. 5 µg membranes, 0.5 mg SPA-beads, 60 pM ^{125}I-tracer (^{125}I-glucagon or ^{125}I-GLP-1), and native glucagon or LAG in increasing concentrations (0-1 µM, diluted in buffer with 50 mM HEPES, 1mM CaCl2, 1mM MgCl2, 0.02% tween20, pH = 7.4) were added to each well of a 384-well plate (final volume 40 µl). The plate was incubated for 2 hours at 25ºC. Then it was centrifuged at 1,500 RPM for 10 minutes before reading in a scintillation counter (Topcounter).

The Adenylyl Cyclase Activation Flashplate® Assay (PerkinElmer #SMP004A) is a scintillation based assay to detect intracellular levels of cAMP. Primary rat hepatocytes were centrifuged and re-suspended in stimulation buffer with IBMX (provided with the kit) (1 x 10^6 cells/mL). 50 µL cell suspension were added to the FlashPlate (50.000 cells/well) and stimulated with 50 µL ligands diluted in PBS with 0.02% Tween-20 and 20 mM glucose. After incubation for 30 min. detection buffer from the kit with radiolabelled cAMP was added and after incubation for 2-24 hours the FlashPlate was read in a Topcounter. The concentration of cAMP produced was interpolated from the standard curve (prepared according to the protocol from the kit).
