# Supplementary material for: Sustained effect of glucagon on body weight and blood glucose: Assessed by continuous glucose monitoring in diabetic rats
Source: PLoS One. 2018 Mar 20;13(3):e0194468. doi: 10.1371/journal.pone.0194468 (PMC5860770; doi:10.1371/journal.pone.0194468)
Supplement: S1 Fig — Mean activity from 0–8 h. after dosing of 86 nmol/kg insulin ± 1 nmol/kg LAG (A) and 120 nmol/kg insulin ± 1 nmol/kg LAG (B) during the dark period. Data are expressed as means of 5 days (86 nmol/kg insulin) and 2 days (120 nmol/kg insulin), respectively ± SEM; n = 4–8. (PDF) [file pone.0194468.s002.pdf]

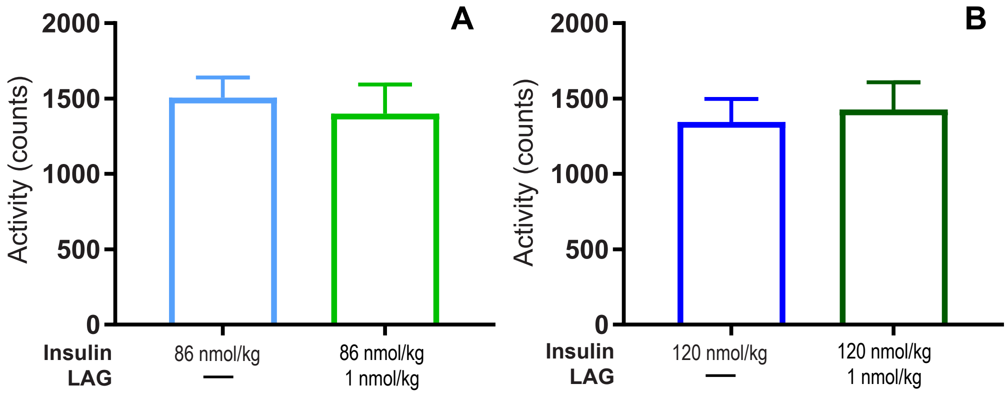

S1 Fig: Glucagon effect on activity during dark period.
Mean activity from 0-8 h. after dosing of 86 nmol/kg insulin ± 1 nmol/kg LAG (A) and 120 nmol/kg insulin ± 1 nmol/kg LAG (B) during the dark period. Data are expressed as means of 5 days (86 nmol/kg insulin) and 2 days (120 nmol/kg insulin), respectively ± SEM; n = 4-8.
