# Supplementary material for: Sustained effect of glucagon on body weight and blood glucose: Assessed by continuous glucose monitoring in diabetic rats
Source: PLoS One. 2018 Mar 20;13(3):e0194468. doi: 10.1371/journal.pone.0194468 (PMC5860770; doi:10.1371/journal.pone.0194468)
Supplement: S2 Fig — Insulin in plasma (nM) 60 min after sc. dosing of 103 nmol/kg insulin ± 1 nmol/kg LAG. Insulin in plasma was measured by an internal developed LOCI assay (see details below). There was no significant difference between the plasma levels of insulin with or without the presence of 1 nmol/kg LAG (p = 0.6, analyzed using a two-tailed unpaired t-test). Data are expressed as means ± SEM; n = 12. (PDF) [file pone.0194468.s003.pdf]

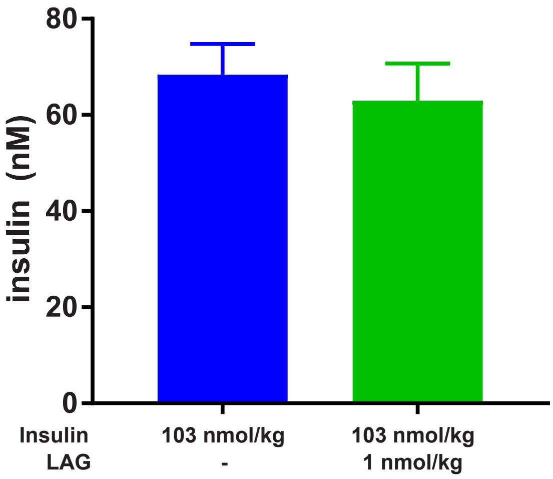

S2 Fig: Insulin in plasma with and without 1 nmol/kg LAG

Insulin in plasma (nM) 60 min after sc. dosing of 103 nmol/kg insulin ± 1 nmol/kg LAG. Insulin in plasma was measured by an internal developed LOCI assay (see details below). There was no significant difference between the plasma levels of insulin with or without the presence of 1 nmol/kg LAG ($p = 0.6$, analyzed using a two-tailed unpaired t-test). Data are expressed as means ± SEM; $n = 12$.

Method

Samples were analyzed for human insulin content using Luminescence Oxygen Channeling Immunoassay (LOCI), which is a homogenous bead based assay. LOCI reagents include two latex bead reagents and a biotinylated antibody, which is one of the antibodies in the sandwich. One of the bead reagents is a generic reagent (donor beads) and is coated with streptavidin and contains a photosensitive dye. The second bead reagent (acceptor beads) is coated with the other antibody making up the sandwich. During the assay the three reactants combine with analyt to form a bead-aggregate-immune complex. Illumination of the complex releases singlet oxygen from the donor beads which channels into the acceptor beads and triggers chemiluminescence which is measured in the EnVision plate reader. The amount of light generated is proportional to the concentration of human insulin. 1 µL sample/calibrator/control is applied in 384-well LOCI plates. 15 µL of a mixture of biotinylated mAb OXI005-03B and mAb HUI-018-conjugated acceptor-beads is added to each well (21-22 ºC). The plates are incubated for 1 h at 21–22 ºC. 30 µL streptavidin coated donorbeads (67 µg/mL) is added to each well and all is incubated for 30 minutes at 21-22 ºC. The plates are read in an Envision plate reader at 21-22 ºC with a filter having a bandwidth of 520-645 nm after excitation by a 680 nm laser. The total measurement time per well is 210 ms including a 70 ms excitation time. The lower limit of quantification is 4 pM. Cross-reactivity to rat insulin is below 1%.
